# Helminth-infected patients with malaria: a low profile transmission hub?

**DOI:** 10.1186/1475-2875-11-376

**Published:** 2012-11-15

**Authors:** Mathieu Nacher

**Affiliations:** 1 Epidémiologie des Parasitoses et Mycoses Tropicales, EA3593, Université des Antilles et de la Guyane, Campus Saint Denis, Cayenne, 97300, Guyane Française; 2 Centre d’Investigation Clinique Epidémiologie Clinique CIE INSERM 802, Centre Hospitalier de Cayenne, Cayenne, Guyane Française

**Keywords:** Malaria, Worms, Coinfection, Immunomodulation, Anemia, Asymptomatic, Transmission, Vector

## Abstract

Eclipsed by the debates about malaria incidence and severity in individual patients, malaria transmission in helminth-infected persons has so far received very little attention. Studies in humans have shown increased malaria incidence and prevalence, and a trend for a reduction of symptoms in patients with malaria. This suggests that such patients could possibly be less likely to seek treatment thus carrying malaria parasites and their gametocytes for longer durations, therefore, being a greater potential source of transmission. In addition, in humans, a study showed increased gametocyte carriage, and in an animal model of helminth-malaria co-infection, there was increased malaria transmission. These elements converge towards the hypothesis that patients co-infected with worms and malaria may represent a hub of malaria transmission. The test of this hypothesis requires verifying, in different epidemiological settings, that helminth-infected patients have more gametocytes, that they have less symptomatic malaria and longer-lasting infections, and that they are more attractive for the vectors. The negative outcome in one setting of one of the above aspects does not necessarily mean that the other two aspects may suffice to increase transmission. If it is verified that patients co-infected by worms and malaria could be a transmission hub, this would be an interesting piece of strategic information in the context of the spread of anti-malarial resistance and the malaria eradication attempts.

## Background

In the past decade, the topic of interactions between worms and malaria has generated a surge of interest with over 35 publications reporting findings in humans from different continents. Similarly, there have been over 25 publications on different animal models of co-infection between worms and malaria [1]. So far, the various study designs used have yielded snapshots of information about specific endpoints but no dynamic view of what happens today or what used to take place in the pre-therapeutic era. Most of the publications on co-infections in humans focussed on whether malaria was more or less frequent or whether malaria was more or less severe in patients with worms. These are important topics, and with time and more and more data points, a pattern now seems to have emerged, with hookworm regularly linked to an increase of malaria and *Ascaris* regularly linked to a decrease in malaria and malaria severity. Hypothetical mechanisms have been put forward: the immunomodulation of the host response on one side, and the increased attractiveness of the anaemic host for the vectors presumably leading to an increase of the number of mosquito bites on the other side. The apparent discrepancies between authors may also have resulted from the very different study designs ranging from cross sectional studies to randomized trials, with exposure to worms considered one species at a time or pooled together. It is also possible that the overlapping environmental envelopes of *Plasmodium* and worms may also have led to some of the observed associations. Hopefully, interest in this topic will be sustained leading to a better understanding of mechanisms which underpin it.

### The hypothesis

Eclipsed by the debates about malaria incidence and severity in individual patients, malaria transmission in helminth-infected persons is an area that has so far received very little attention. The present hypothesis is mostly constructed from the available studies in humans, but also from animal models. It attempts to connect observations of variable reliability given the study designs used to obtain the information. In this hypothesizing exercise, refraining from attempting to connect the dots in order to avoid type 1 errors (wrongly rejecting the status quo) would be a mistake. It is just a hypothesis, and to be tested. If it is false then nothing changes, but if it is true the implications would not be trivial. The hypothesis may appear piecemeal, but the general idea is that the various observations made so far seem to converge towards the ultimate amplification of transmission.

In 2001, cross-sectional data from Thailand showed a two-fold increase in gametocyte carriage linked to decreased haemoglobin in helminth-infected patients [2]. There have been so far no attempts to replicate these findings in humans elsewhere. Furthermore, an animal model using BALB/C mice infected with *Echinostoma caproni* and *Plasmodium yoelii* showed enhanced transmission with a two-fold increase of transmission to mosquitoes fed on co-infected mice relative to mice with *Plasmodium* only [3]. Despite the potential epidemiological importance of these findings research remains focussed on more obvious clinical aspects. Arguably though, the pieces of the puzzle of knowledge accumulated so far in humans also points towards the question of transmission. Some of the findings suggested there was an increase in incidence [4-8], and other studies pointed towards an increase in prevalence [9-11]. Prevalence (P), the number of persons with malaria at a point in time is influenced on the one hand by the incidence (I), the number of new infections and on the other hand by the duration (D) of these infections (patients may get cured, or may die). The classic formula P=IxD implies that prevalence will increase if incidence increases and /or if infection duration increases.

The cross-sectional studies finding increased malaria prevalence among helminth-infected patients could not determine whether prevalence was higher because incidence was higher or whether prevalence was higher because malaria lasted longer in helminth-infected patients, or both. Some studies have definitely shown increased incidence of malaria in helminth-infected patients. No studies have looked if malaria lasted longer in helminth-infected patients. However, there are converging elements that point towards such a possibility. The general trend so far was that malaria was less “noisy” in helminth-infected patients: less fever [12], lower parasitaemia [13-15], less severe symptoms [16-18], and in some cases less anaemia [19,20]. In some of the above studies malaria was asymptomatic and more frequent in helminth-infected patients. Other studies showed that anthelmintics exacerbated malaria. The study by Murray *et al*. showed that piperazine treatment of *Ascaris*-infected patients led to an early increase (6–14 days) of malaria suggesting that the parasites had been there all along without causing clinical malaria [21]. In poor tropical areas, healthcare is often sought at very advanced stages of disease, it is reasonable to wonder whether these helminth-infected patients with fewer symptoms wait more to consult, or even never consult while all along they carry malaria parasites and their gametocytes for prolonged periods of time.

### Testing the hypothesis

The transmission hypothesis is a combination of different components: the increased in gametocyte carriage, the increased duration of infection, and the increased attractivity of the host for the vector. A first step, possibly very easy to test by re-analysing available data would be to verify whether gametocyte carriage is affected by worms. Another step would be to determine whether humans with worms are really more attractive for vectors, and to look at the importance haemoglobin concentration as a proximal determinant. Finally, the bottom of the malaria iceberg represented by asymptomatic *Plasmodium* infections should be described in relation to worms using sensitive methods. Testing the hypothesis that the duration of infection increases in patients with worms would require observing patients with asymptomatic malaria longitudinally, without treating them, which may pose ethical problems and thus be difficult to test in humans.

The hypothesis seems rather robust since the local or general refutation of one of these three aspects would not necessarily imply that malaria has no effect on transmission because the other two could still be operating.

### The implications

Whether the mechanism of pauci-symptomatic malaria in patients with worms is immunological or linked to nutritional consequences of worms [22], or both, remains to be seen. If this scenario is correct, then helminth-infected patients could constitute a major source of transmission. Furthermore, if it is confirmed that they are also more likely to harbour circulating gametocytes, and to attract mosquitoes as they become anaemic, the transmission potential could be even higher. Thus, the increase in incidence that seems linked to hookworm and the reduction of morbidity and severity mostly observed with *Ascaris* converges towards the creation of a smouldering reservoir for prolonged malaria transmission. The increased incidence and prevalence, combined with some observations found increased multiplicity of infection [23], and increased mixed *vivaxfalciparum* co-infections in patients with worms [24] suggest that the diversity of parasites could be greater in helminth-infected patients. This could theoretically speed the acquisition of premunition in individuals. In addition, the increased genetic diversity of the oocysts in the mosquitoes having fed on coinfected patients could theoretically lead to a more genetically diverse malaria parasites circulating in the population. The evolutionary implications of co-infections could also extend to the reproduction of worms, which have an interest in protecting their host to survive and reproduce [25].

In a given place, what proportion of transmission is attributable to asymptomatic or pauci-symptomatic patients is not known. At a time when malaria eradication is discussed and drug resistance is spreading, it seems important to test the possibility that helminth-infected populations, notably by a combination of hookworm and *Ascaris*, could constitute a transmission hub for malaria parasites. The hookworm vaccine aimed at reducing the loss of haemoglobin could thus have unforeseen benefits.

## Competing interests

The authors declare that they have no competing interests.

## References

[B1] Nacher M Interactions between worms and malaria: good worms or bad worms? Malar J 2011 10 259 10.1186/1475-2875-10-259 21910854 PMC3192711

[B2] Nacher M Singhasivanon P Silachamroon U Treeprasertsu S Krudsood S Gay F Mazier D Looareesuwan S Association of helminth infections with increased gametocyte carriage during mild falciparum malaria in Thailand Am J Trop Med Hyg 2001 65 644 647 11716129 10.4269/ajtmh.2001.65.644

[B3] Noland GS Graczyk TK Fried B Kumar N Enhanced malaria parasite transmission from helminth co-infected mice AmJTrop Med Hyg 2007 76 1052 1056 17556610

[B4] Nacher M Singhasivanon P Yimsamran S Manibunyong W Thanyavanich N Wuthisen R Looareesuwan S Intestinal helminth infections are associated with increased incidence of Plasmodium falciparum malaria in Thailand J Parasitol 2002 88 55 58 12053980 10.1645/0022-3395(2002)088[0055:IHIAAW]2.0.CO;2

[B5] Spiegel A Tall A Raphenon G Trape JF Druilhe P Increased frequency of malaria attacks in subjects co-infected by intestinal worms and Plasmodium falciparum malaria Trans R Soc Trop Med Hyg 2003 97 198 199 10.1016/S0035-9203(03)90117-9 14584377

[B6] Sokhna C Le Hesran JY Mbaye PA Akiana J Camara P Diop M Ly A Druilhe P Increase of malaria attacks among children presenting concomitant infection by schistosoma mansoni in Senegal Malar J 2004 3 43 10.1186/1475-2875-3-43 15544703 PMC538284

[B7] Roussilhon C Brasseur P Agnamey P Perignon JL Druilhe P Understanding human-plasmodium falciparum immune interactions uncovers the immunological role of worms PLoS One 2010 5 e9309 10.1371/journal.pone.0009309 20174576 PMC2824820

[B8] Adegnika AA Ramharter M Agnandji ST Ateba Ngoa U Issifou S Yazdanbahksh M Kremsner PG Epidemiology of parasitic co-infections during pregnancy in lambarene, Gabon Trop Med Int Health 2010 15 1204 1209 10.1111/j.1365-3156.2010.02598.x 20636299

[B9] Hillier SD Booth M Muhangi L Nkurunziza P Khihembo M Kakande M Sewankambo M Kizindo R Kizza M Muwanga M Elliott AM Plasmodium falciparum and helminth coinfection in a semi urban population of pregnant women in Uganda J Infect Dis 2008 198 920 927 10.1086/591183 18721060 PMC2886962

[B10] Midzi N Sangweme D Zinyowera S Mapingure MP Brouwer KC Munatsi A Mutapi F Mudzori J Kumar N Woelk G Mduluza T The burden of polyparasitism among primary schoolchildren in rural and farming areas in Zimbabwe Trans R Soc Trop Med Hyg 2008 102 1039 1045 10.1016/j.trstmh.2008.05.024 18656215

[B11] Yatich NJ Yi J Agbenyega T Turpin A Rayner JC Stiles JK Ellis WO Funkhouser E Ehiri JE Williams JH Malaria and intestinal helminth co-infection among pregnant women in Ghana: prevalence and risk factors Am J Trop Med Hyg 2009 80 896 901 19478245

[B12] Nacher M Singhasivanon P Traore B Dejvorakul S Phumratanaprapin W Looareesuwan S Gay F Short report: hookworm infection is associated with decreased body temperature during mild plasmodium falciparum malaria Am J Trop Med Hyg 2001 65 136 137 11508388 10.4269/ajtmh.2001.65.136

[B13] Briand V Watier L JY LEH Garcia A Cot M Coinfection with Plasmodium falciparum and Schistosoma haematobium: protective effect of schistosomiasis on malaria in senegalese children? Am J Trop Med Hyg 2005 72 702 707 15964953

[B14] Brutus L Watier L Hanitrasoamampionona V Razanatsoarilala H Cot M Confirmation of the protective effect of ascaris lumbricoides on plasmodium falciparum infection: results of a randomized trial in Madagascar Am J Trop Med Hyg 2007 77 1091 1095 18165528

[B15] Degarege A Animut A Legesse M Erko B Malaria severity status in patients with soil-transmitted helminth infections Acta Trop 2009 112 8 11 10.1016/j.actatropica.2009.05.019 19497286

[B16] Nacher M Gay F Singhasivanon P Krudsood S Treeprasertsuk S Mazier D Vouldoukis I Looareesuwan S Ascaris lumbricoides infection is associated with protection from cerebral malaria Parasite Immunol 2000 22 107 113 10.1046/j.1365-3024.2000.00284.x 10672191

[B17] Nacher M Singhasivanon P Silachamroon U Treeprasertsuk S Vannaphan S Traore B Gay F Looareesuwan S Helminth infections are associated with protection from malaria-related acute renal failure and jaundice in Thailand Am J Trop Med Hyg 2001 65 834 836 11791982 10.4269/ajtmh.2001.65.834

[B18] Nacher M Singhasivanon P Traore B Vannaphan S Gay F Chindanond D Franetich JF Mazier D Looareesuwan S Helminth infections are associated with protection from cerebral malaria and increased nitrogen derivatives concentrations in Thailand Am J Trop Med Hyg 2002 66 304 309 12139225 10.4269/ajtmh.2002.66.304

[B19] Melo GC Reyes-Lecca RC Vitor-Silva S Monteiro WM Martins M Benzecry SG Alecrim MG Lacerda MV Concurrent helminthic infection protects schoolchildren with plasmodium vivax from anemia PLoS One 2010 5 e11206 10.1371/journal.pone.0011206 20574512 PMC2888569

[B20] Kung’u JK Goodman D Haji HJ Ramsan M Wright VJ Bickle QD Tielsch JM Raynes JG Stoltzfus RJ Early helminth infections are inversely related to anemia, malnutrition, and malaria and are not associated with inflammation in 6- to 23-month-old Zanzibari children Am J Trop Med Hyg 2009 81 1062 1070 10.4269/ajtmh.2009.09-0091 19996438

[B21] Murray J Murray A Murray M Murray C The biological suppression of malaria: an ecological and nutritional interrelationship of a host and two parasites Am J Clin Nutr 1978 3 1363 1366 354372 10.1093/ajcn/31.8.1363

[B22] Nacher M McGready R Stepniewska K Cho T Looareesuwan S White NJ Nosten F Haematinic treatment of anaemia increases the risk of Plasmodium vivax malaria in pregnancy Trans R Soc Trop Med Hyg 2003 97 273 276 10.1016/S0035-9203(03)90140-4 15228240

[B23] Chaorattanakawee S Natalang O Hananantachai H Nacher M Brockman A Nosten F Looareesuwan S Patarapotikul J Trichuris trichiura infection is associated with the multiplicity of plasmodium falciparum infections, in Thailand Ann Trop Med Parasitol 2003 97 199 202 10.1179/000349803125002968 12803876

[B24] Nacher M Singhasivanon P Gay F Silachomroon U Phumratanaprapin W Looareesuwan S Contemporaneous and successive mixed plasmodium falciparum and plasmodium vivax infections are associated with ascaris lumbricoides: an immunomodulating effect? J Parasitol 2001 87 912 915 11534659 10.1645/0022-3395(2001)087[0912:CASMPF]2.0.CO;2

[B25] Nacher M Singhasivanon P Krudsood S Phumratanaprapin W Vanaphan S Tangpukdee N Carme B Looareesuwan S Inverse relationship between the number of fertilized ascaris eggs excreted and fever, in patients coinfected with plasmodium vivax and ascaris lumbricoides Ann Trop Med Parasitol 2005 99 623 625 10.1179/136485905X51364 16156977

